# A Self-Consistent Phase Field Crystal Method for Twisted Bilayer Graphene

**DOI:** 10.3390/nano16161000

**Published:** 2026-08-14

**Authors:** Pingqia Wang, Kai Liu

**Affiliations:** Faculty of Arts and Sciences, Beijing Normal University at Zhuhai, No. 18 Jinfeng Road, Zhuhai 519087, China

**Keywords:** bilayer graphene, generalized stacking-fault energy, phase field method

## Abstract

Correlated electronic phenomena in magic-angle twisted bilayer graphene have garnered widespread research interest in two-dimensional materials science. As a powerful multiscale framework bridging atomic-scale resolution and mesoscopic structural evolution, the structural phase field crystal method has been widely adopted for graphene system studies. In this work, we develop a self-consistent XPFC model specifically for twisted bilayer graphene (tBLG) simulations. By globally optimizing the core free-energy functional parameters via a genetic algorithm, the proposed model achieves a marked improvement in consistency between the equilibrium density field and the first-principles generalized stacking fault energy surface. We further introduce a self-consistent dynamic interlayer interaction potential to replace the conventional fixed-substrate approximation, which captures the bidirectional coupling and mutual relaxation between adjacent graphene layers in a self-consistent manner. We calibrate the precise magnitude of the interlayer potential using the widths of stacking domain boundaries between distinct stacking configurations as a key metric, with the results benchmarked against atomistic simulation data. When applied to the 1.1° magic-angle tBLG system, the model uncovers spontaneous structural relaxation driven by interlayer van der Waals interactions: low-energy AB–BA stacking domains expand significantly, while high-energy AA domains shrink correspondingly.

## 1. Introduction

Graphene, a single layer of carbon atoms tightly packed into a hexagonal honeycomb lattice, stands as one of the most revolutionary two-dimensional (2D) materials discovered to date [1]. Its exceptional mechanical, electrical, and thermal properties have opened up new frontiers in condensed matter physics and materials science [2,3,4]. Unlike single-layer graphene, bilayer graphene can exist in multiple stacking configurations—most notably Bernal (AB/BA) stacking and twisted stacking—each exhibiting distinct physical behaviors [5,6,7]. A landmark breakthrough came in 2018 when Cao et al. demonstrated that twisted bilayer graphene (tBLG) exhibits unconventional superconductivity near the first magic angle (∼1.1°) [8], a discovery that triggered an unprecedented global research surge into moiré superlattices and correlated electronic phases in 2D heterostructures [9,10]. Recent experiments have further expanded this frontier, demonstrating exotic phenomena such as fractional Chern insulators [11], emergent localized moments [12], and optical control of orbital magnetism [13]. Continuum relaxation models and moiré elasticity studies systematically reveal the lattice deformation, strain field distribution and atomic reconstruction behaviors in tBLG, establishing the fundamental mechanical basis for understanding moiré superlattice stabilization [14,15,16]. Machine learning interatomic potentials provide high-fidelity descriptions of interlayer atomic interactions in tBLG systems [17].

Computational modeling plays an important role in unraveling the complex structural and electronic properties of graphene systems, especially those that are challenging to characterize experimentally [18]. On the continuum scale, the phase field crystal (PFC) method has emerged as a powerful multiscale modeling technique that bridges atomic-scale details and mesoscale microstructure evolution. Unlike traditional continuum methods, the PFC approach describes phase-transformation dynamics through an atomically resolved order-parameter field that is loosely coupled to the atomic density field, enabling the simulation of long-time-scale processes while retaining atomic-level precision. The original PFC model was initially developed for 2D triangular lattices and 3D cubic crystal symmetries [19,20] and has since been widely applied to study grain boundary formation, dislocation dynamics, and crystal growth. In recent years, extensions of the PFC method have been successfully applied to graphene systems, including the simulation of dendritic graphene growth via anisotropic surface diffusion [21,22,23]. A critical advancement was the development of the structural phase field crystal (XPFC) model, which incorporates a rotationally invariant three-point correlation function into the excess free-energy functional [24,25]. This innovation enables the XPFC model to accurately capture the hexagonal lattice symmetry of graphene, as well as its atomic-scale defects, nucleation kinetics, and diffusional growth from disordered precursor phases [26,27].

Despite these advances, conventional XPFC models for multilayer graphene still suffer from three key limitations that hinder their predictive capability. First, the density field distribution generated by standard XPFC parameters deviates significantly from the first-principles generalized stacking fault energy (GSFE) surface, leading to inaccurate descriptions of interlayer van der Waals interactions [26,28,29,30]. Second, traditional XPFC implementations often suffer from poor numerical stability, which requires small time steps and results in prohibitively high computational costs for large-domain simulations. Third, most previous studies adopt a fixed-bottom-layer assumption, treating the lower graphene layer as a rigid substrate [27]. This approximation neglects the dynamic mutual relaxation between adjacent layers, which is important for accurately modeling structural evolution in twisted and multilayer systems.

In this paper, we address these limitations by developing a self-consistent XPFC model for bilayer graphene systems that can be straightforwardly extended to multilayer systems. Our main contributions are threefold. First, we employ a genetic algorithm to systematically optimize the free-energy parameters of the XPFC model [31]. The self-consistent model yields a density field distribution that achieves an approximately 26-fold reduction in deviation from the first-principles GSFE surface compared to the conventional model while also dramatically improving numerical stability and computational efficiency. Second, we calibrate the strength of the interlayer potential using a long narrow bilayer graphene ribbon domain and demonstrate that the stacking-fault-energy bandwidth obtained from our model shows superior agreement with atomistic simulation results compared to the traditional GSFE-based approach [27,28]. Third, we introduce a dynamically evolving interlayer interaction potential that is self-consistently generated from the phase field of each adjacent layer. Unlike the fixed-substrate approximation, this approach captures the mutual coupling and relaxation between all layers, enabling the simulation of arbitrary multilayer graphene [32]. We then apply our optimized XPFC model to investigate the structural relaxation of magic-angle tBLG at 1.1°, using the supercell method to construct the moiré superlattice [33]. Our findings show that bilayer relaxation gives rise to an increased fraction of low-energy AB/BA stacking and a contracted area of high-energy AA stacking via in-plane atomic displacement [34].

The remainder of this paper is organized as follows. In Section 2, we present the mathematical formulation of the self-consistent XPFC model. Section 3 describes the numerical results, including the simulation results for magic-angle tBLG. Finally, in Section 4, we summarize our key findings and discuss future directions.

## 2. Modeling and Methods

We consider a bilayer graphene system, with the spatial phase density of carbon atoms for the *i*-th layer (i=1,2) described by $\rho_{i}$. A dimensionless density field $\psi_{i}$ is defined to normalize the local density deviation from the reference density of the disordered phase $\psi_{i}=\frac{\rho_{i}-{\bar{\rho }}_{i}}{{\bar{\rho }}_{i}}$, where ${\bar{\rho }}_{i}$ is the reference density of the disordered phase for the *i*-th layer. The total free energy of the crystallization system is formulated as

(1) $$F_{total,i}=F_{id}\left(\psi_{i}\right)+f_{2}F_{ex,2}\left(\psi_{i}\right)+f_{3}F_{ex,3}\left(\psi_{i}\right)+F_{IL}\left(\psi_{i}\right),$$

where $F_{id}$ is the ideal free energy, $F_{ex,2}$ is the two-point interaction energy, $F_{ex,3}$ is the three-point correlation energy, and $F_{IL}$ is the adjacent layer interaction potential. Here, $f_{2}$ and $f_{3}$ are dimensionless coefficients representing the strength of the corresponding energy terms, which are calibrated along with other core parameters via the genetic algorithm detailed in Section 2.3.

### 2.1. XPFC Free-Energy Functional Terms

The ideal free energy $F_{id}$ takes the following dimensionless form:

(2) $$F_{id}=\int dx\left(\frac{\psi_{i}^{2}}{2}-\eta \frac{\psi_{i}^{3}}{6}+\chi \frac{\psi_{i}^{4}}{12}\right),$$

where η and χ are dimensionless parameters to adjust the form of the ideal free energy and to be determined later by the genetic algorithm together with $f_{2}$, $f_{3}$, and the average phase density ${\bar{\psi }}_{i}$.

The two-point correlation energy is based on hard-sphere-like interactions and is governed by

(3) $$F_{ex,2}=-\frac{1}{2}\int \psi_{i}\left(x\right)\int C_{2}\left(x-x^{\prime }\right)\psi_{i}\left(x^{\prime }\right)dx^{\prime }dx,$$

where $C_{2}$ is the two-point correlation function, a real-space circularly symmetric cutoff function designed to exclude non-physical long-range correlations. It takes the form

(4) $$C_{2}\left(x\right)=-\frac{R}{\pi r_{0}^{2}}\mathrm{circ}\left(\frac{r}{r_{0}}\right),\;\mathrm{with}\;\mathrm{circ}\left(r\right)=\left\{\begin{array}{cc} 1, & r\leq 1\\ 0, & r>1 \end{array}\right.$$

where $r_{0}$ is the cutoff radius for the repulsive term, r=∥x∥, and *R* is the magnitude of the repulsion.

The three-point correlation energy $F_{ex,3}$ is the key innovation that distinguishes the XPFC model from conventional PFC extensions as it is rotationally invariant and sufficiently robust to capture a number of 2D crystal structures described by a single bond angle [25,27]. It is governed by

(5) $$F_{ex,3}\left(\psi_{i}\right)=-\frac{1}{3}\int \psi_{i}\left(x\right)\int C_{3}\left(x-x^{\prime },x-x^{\prime \prime }\right)\psi_{i}\left(x^{\prime }\right)\psi_{i}\left(x^{\prime \prime }\right)dx^{\prime }dx^{\prime \prime }dx,$$

where the three-point correlation function $C_{3}$ is defined as

(6) $$C_{3}\left(x-x^{\prime },x-x^{\prime \prime }\right)=\sum_{i}C_{s}^{\left(k\right)}\left(x-x^{\prime }\right)C_{s}^{\left(k\right)}\left(x-x^{\prime \prime }\right),$$

and, in polar coordinates x=(x,y)=(rcosθ,rsinθ), the function $C_{s}^{\left(k\right)}$ reads as

(7) $$C_{s}^{\left(1\right)}\left(r,\theta \right)=C_{r}\left(r\right)cos\left(m\theta \right),\;C_{s}^{\left(2\right)}\left(r,\theta \right)=C_{r}\left(r\right)sin\left(m\theta \right),\;C_{r}\left(r\right)=\frac{X}{2\pi a_{0}}\delta \left(r-a_{0}\right),$$

where *X* is a parameter defining the interaction strength, $a_{0}$ corresponds to the lattice constant satisfying $r_{0}/a_{0}=1.22604$, and m=3 defines the bond order of the crystal phase. For the graphene system, we take R=6 and $X^{-1}=0.4$ [25,27].

### 2.2. Self-Consistent Dynamic Interlayer Interaction Potential

To address the limitation of the fixed-substrate approximation, we design a dynamically evolving interlayer interaction potential $F_{IL}$ that is determined by the real-time density fields of both adjacent layers. The physical origin of this potential lies in the coupling of interlayer van der Waals (vdW) interactions at the moiré scale, which have been shown to be the primary driving force behind the structural relaxation of tBLG [16,35]. The potential takes the following form:

(8) $$F_{IL}=\int V_{BL}\left(x\right)\psi_{i}\left(x\right)dx,$$

where $V_{BL}\left(x\right)$ is the effective stacking energy potential generated by the adjacent layer. In this work, we compare two different formulations of $V_{BL}\left(x\right)$:

**Traditional GSFE-Based Potential:** This formulation uses a precomputed fixed potential field derived from the first-principles GSFE surface of a rigid graphene substrate. It is defined as $V_{BL}\left(x\right)=\frac{1}{\lambda_{F}}F_{\mathrm{GSFE}}\left(x\right)$, where $F_{\mathrm{GSFE}}\left(x\right)$ is the analytical GSFE function fitted to first-principles data and $\lambda_{F}$ is a dimensionless calibration parameter that absorbs the dimensional conversion between the GSFE energy scale and the dimensionless free-energy framework of the XPFC model. The detailed analytical expression of $F_{\mathrm{GSFE}}\left(x\right)$ is provided in the Supplementary Materials.
**Dynamic Self-Consistent Potential**: This new formulation, the core of our methodological improvement, replaces the fixed GSFE potential with a real-time potential field directly generated by the adjacent layer’s density field $V_{BL}\left(x\right)=\frac{1}{\lambda_{\psi }}{\tilde{\psi }}_{j}\left(x\right)$ (j≠i), where ${\tilde{\psi }}_{j}\left(x\right)$ is the density field of the *j*-th layer. Here, $\lambda_{\psi }$ is a dimensionless coupling coefficient that calibrates the strength of the interlayer interaction, matching the characteristic length scale of interlayer vdW interactions (∼1 nm).

In this dynamic potential framework, the two layers are coupled bidirectionally: the potential on each layer evolves with the density of the adjacent layer during relaxation. Unlike the fixed-substrate approximation, this approach includes mutual screening and interlayer force coupling between the two graphene layers. Section 3 shows that this feature is important to produce the structural relaxation of tBLG.

### 2.3. Genetic Algorithm Parameter Optimization

The XPFC model’s predictive accuracy depends on matching its free-energy landscape to the first-principles GSFE surface. Five parameters require optimization: the average dimensionless density $\bar{\psi }$, the ideal free-energy coefficients η and χ, and the excess energy strength coefficients $f_{2}$ and $f_{3}$. These are denoted as $c=(\bar{\psi },\eta ,\chi ,f_{2},f_{3})$.

**Figure 1:**
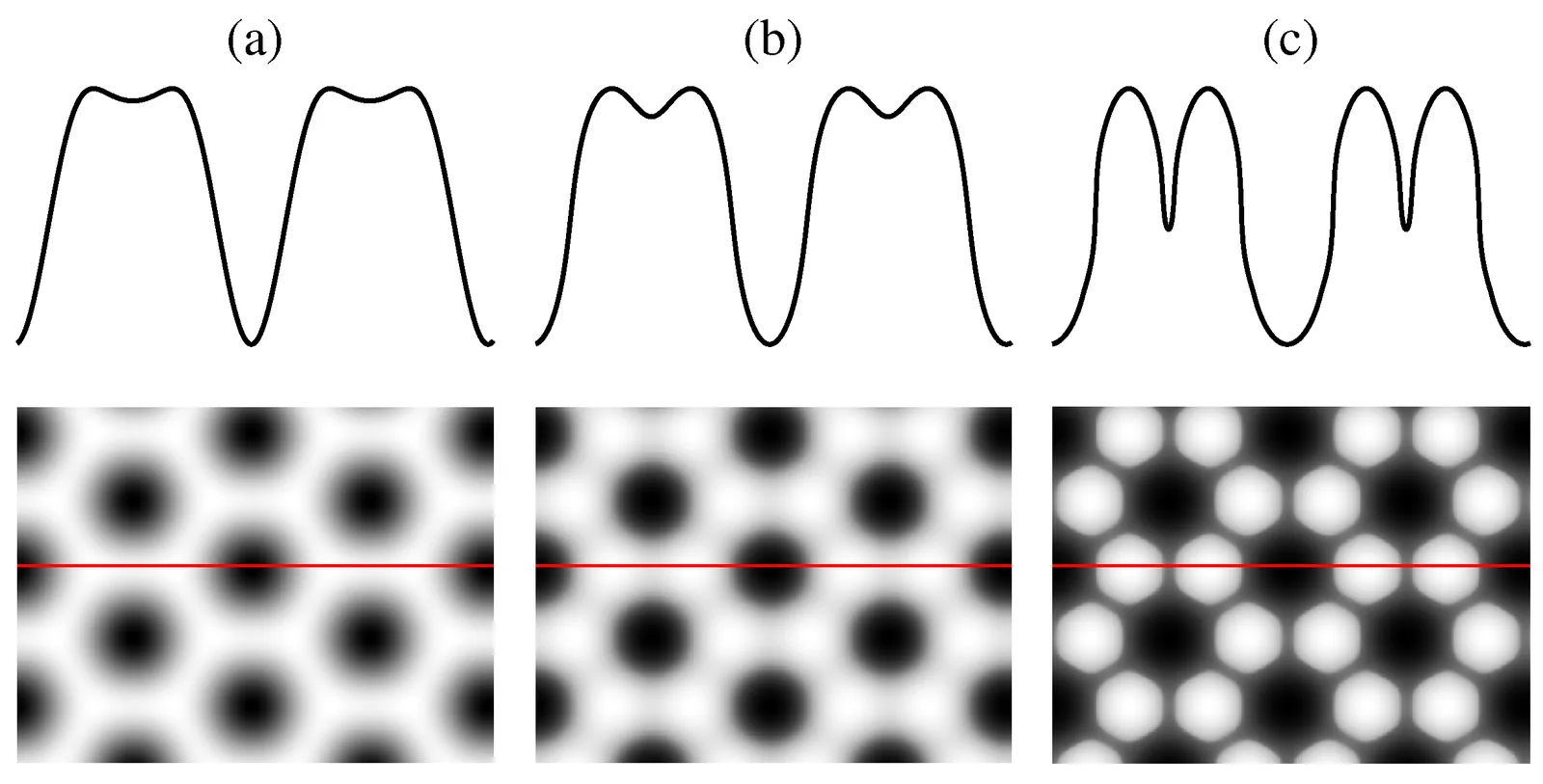
The appearance of PFC density fields in equilibrium: (**a**) GSFE, (**b**) self-consistent XPFC, and (**c**) original XPFC. All density fields have been linearly mapped to grayscale values, with the maxima (minima) appearing as white (black). Density profiles along the red lines that intersect local maxima and minima are shown on top in arbitrary units.

A multi-objective genetic algorithm (GA) was used for calibration. The workflow and key hyperparameters of the GA optimization are as follows:

**Initialization**: The algorithm starts with an initial population of 40 randomly generated parameter sets. The value range for each parameter is determined by the physical and computational characteristics of the XPFC model; e.g., η>0, χ>0, $f_{2}>0$. Specifically, the ranges are $\bar{\psi }\in \left[-0.2,0.5\right]$, η∈[0.1,5], χ∈[0.1,5], $f_{2}\in \left[0.1,5\right]$, $f_{3}\in \left[0.1,5\right]$. It is desirable to avoid excessive excursions of any individual quantity as such values would be intuitively unphysical. The initial guess is set to $c_{0}=\left(0.3,1,1,1,1\right)$, consistent with the original XPFC model setup.
**Evaluation and fitness calculation**: For each parameter set, a single-layer XPFC simulation is run to steady state. Fitness is evaluated via a weighted function that measures the $L^{2}$ norm of the difference between the XPFC equilibrium density profile and the GSFE reference profile. That is to say, the genetic algorithm solves the following constrained optimization problem:
  (9) $$\min_{c\in U}\epsilon \;,\;\;\;\mathrm{where}\;\epsilon \left(c\right)=\int_{L}{\left(\tilde{\psi }\left(x,c\right)-{\tilde{F}}_{GSFE}\left(x\right)\right)}^{2}dx.$$
  Here $U\subset R^{5}$ is the feasible domain, ${\tilde{F}}_{GSFE}$ is the corresponding normalized profile of the GSFE field (the values are linearly rescaled such that the global maximum maps to 1 and the global minimum maps to 0), as shown in Figure 1a, and $\tilde{\psi }\left(x,c\right)$ is the normalized profile of the single-layer steady-state density field.
**Selection, crossover, and mutation**: Tournament selection (tournament size 3) selects parents. Simulated binary crossover (probability 0.7) and polynomial mutation (probability 0.1) generate offspring.
**Termination criterion**: Iteration stops when the fitness falls below $10^{-6}$ or after 1000 generations, whichever occurs first.

The optimization ran for about two weeks on a high-performance computing server and converged to $c_{m}$. This set reduces the objective function from $\epsilon \left(c_{0}\right)=1.7\times 10^{-2}$ (original model) to $\epsilon \left(c_{m}\right)=6.4\times 10^{-4}$ (self-consistent model), a factor of ∼26. As shown in Figure 1, the self-consistent XPFC model reproduces the spatial distribution of the GSFE field more closely than the original model.

### 2.4. Numerical Solution Strategy and Computational Efficiency

The evolution of the conserved density field $\psi_{i}$ is governed by the dissipative phase field crystal dynamic equation,

(10) $$\frac{\partial \psi_{i}}{\partial t}=M_{\psi_{i}}\nabla^{2}\left(\frac{\delta F_{total,i}}{\delta \psi_{i}}\right)$$

where $M_{\psi_{i}}$ is an effective mobility. For consistency with previous studies [25,27], we set $M_{\psi_{i}}=1$ in all simulations.

Let ${\hat{\psi }}_{i}\left(k,t\right)$ denote the Fourier transform of the real-space density field $\psi_{i}\left(x,t\right)$, where $k=(k_{x},k_{y})$ is the dimensionless wave vector. For the dimensionless computational setting corresponding to Δx=Δy=1, the multiplier simplifies to $-{\hat{\nabla }}^{2}\left(k\right)=4-2\cos k_{x}-2\cos k_{y}$, converting the spatial differential operator into an algebraic operation that is easy to handle numerically.

Using first-order backward Euler for the implicit linear term and first-order forward Euler for the nonlinear explicit term, the semi-implicit iterative equation in Fourier space is derived as follows:

(11) $$\frac{{\hat{\psi }}_{i}^{n+1}-{\hat{\psi }}_{i}^{n}}{\Delta t}=-M_{\psi_{i}}\cdot {\hat{\nabla }}^{2}\left(k\right)\cdot \left[{\hat{\psi }}_{i}^{n+1}+{\left(\frac{\hat{\delta F_{\mathrm{total},i}}}{\delta \psi_{i}}\right)}^{n}-{\hat{\psi }}_{i}^{n}\right],$$

where ${\hat{\psi }}_{i}^{n}$ is the Fourier-space density field at the *n*-th time step, Δt is the dimensionless time step, *k*, and ${\left(\frac{\hat{\delta F_{\mathrm{total},i}}}{\delta \psi_{i}}\right)}^{n}$ is the Fourier transform of the total free-energy derivative calculated explicitly from the real-space field at the *n*-th step. Rearranging the terms to solve for the density field at the next time step ${\hat{\psi }}_{i}^{n+1}$, we obtain the final update rule:

(12) $${\hat{\psi }}_{i}^{n+1}=\frac{{\hat{\psi }}_{i}^{n}}{1+M_{\psi_{i}}{\hat{\nabla }}^{2}\left(k\right)\Delta t}-\frac{M_{\psi_{i}}{\hat{\nabla }}^{2}\left(k\right)\Delta t}{1+M_{\psi_{i}}{\hat{\nabla }}^{2}\left(k\right)\Delta t}\left[{\left(\frac{\hat{\delta F_{\mathrm{total},i}}}{\delta \psi_{i}}\right)}^{n}-{\hat{\psi }}_{i}^{n}\right]$$

Note that $\left[1+M_{\psi_{i}}{\hat{\nabla }}^{2}\left(k\right)\Delta t\right]\geq 0$. More details are referred to in the Supplementary Materials, including the non-dimensionalization.

The dynamics are solved by a pseudo-spectral method based on the fast Fourier transform (FFT). By converting convolutions in the free-energy functional to element-wise multiplications in Fourier space, the computational cost is reduced from $O(N^{2})$ to O(NlogN). To address the high computational cost of large-domain tBLG simulations, we reimplemented the solver in CUDA C/C++. We conducted a systematic performance benchmark to quantitatively verify the computational efficiency improvement of the modified solver. The test configuration is as follows:

**GPU Solver Test Bed**: NVIDIA Geforce 3090 24GB GPU; CUDA Toolkit 11.0, from NVIDIA Corporation, Santa Clara, CA, United States.
**CPU Solver Test Bed**: AMD Ryzen 7 5700X desktop processor, from Advanced Micro Devices, Inc., Sunnyvale, CA, United States; with MATLAB 2021a, from The MathWorks, Inc, Natick, MA, United States; original pseudo-spectral solver with the same numerical precision and physical parameters.
**Benchmark Case**: 1.1° magic-angle tBLG, computational domain size 4096×4096 grid points, $10^{5}$ steps, and periodic boundary conditions applied in both in-plane directions.
The CPU version required 41.7 h, whereas the GPU version finished in 21 min, yielding a 115× speedup. The acceleration is mainly attributed to alleviating the FFT bottleneck. For the GPU-accelerated solver, the dominant bottleneck is global memory bandwidth. Double-precision floating-point arithmetic capacity remains abundant. In contrast, the CPU-based solver is fundamentally compute-bound, with its performance limited by the CPU’s double-precision FLOPs.

## 3. Numerical Results

### 3.1. Model Calibration and Validation

We calibrated the interlayer potential strengths $\lambda_{F}$ and $\lambda_{\psi }$ using a long narrow bilayer graphene ribbon domain following the validation framework of Refs. [27,28,36]. The calibration targets the steady-state width of the AB–BA stacking domain boundary—a key structural parameter governed by the competition between interlayer van der Waals forces, which favor stacking-order expansion, and in-plane energy, which opposes lattice distortion. Because the domain boundary width is sensitive to the interlayer potential, it serves as a reasonable metric for model validation; further details are provided in the Supplementary Materials.

**Figure 2:**
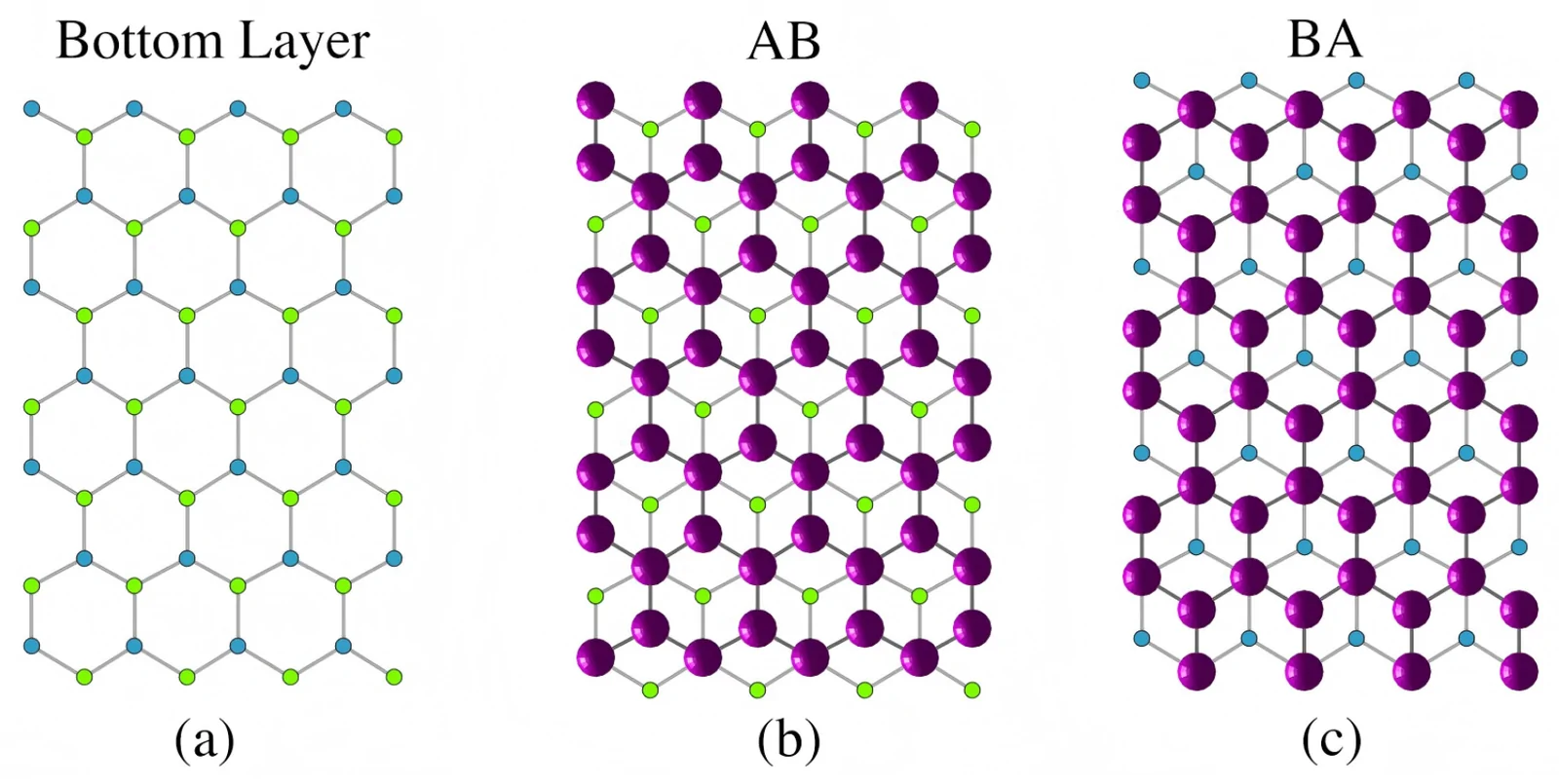
(**a**) Bottom-layer atoms are divided into two groups, with light green and dark green representing different sublattices; (**b**) atomic positions for AB stacking order; (**c**) atomic positions for BA stacking order.

We first precompute the phase fields for Bernal (AB and BA) stacking configurations, denoted by $\psi_{1}\left(x\right)$, $\psi_{2,\mathrm{AB}}\left(x\right)$, and $\psi_{2,\mathrm{BA}}\left(x\right)$, respectively. A well-ordered initial condition is used to generate the bottom-layer phase field $\psi_{1}\left(x\right)$, which enforces periodic boundary conditions on both sides of the rectangular domain (with aspect ratio $L_{1}:L_{2}=2:\sqrt{3}$) and aligns all the hexagonal lattices along the same direction, consistent with Ref. [27]. The initial condition for the top layer $\psi_{2}\left(x,0\right)$ is set to a constant value of $\bar{\psi }=0.2343$, with a small Gaussian noise perturbation. Both AB and BA stacking domains emerge in the simulations, as illustrated in Figure 2. Then, the long bilayer ribbon domain is constructed by concatenating these precomputed phase fields.

The long ribbon is initialized as follows. The bottom layer (L1) is formed by tiling 64 identical patches of the precomputed $\psi_{1}\left(x\right)$ (each with aspect ratio 2:$\sqrt{3}$) side by side. The top layer (L2) has a static initial setup consisting of four parallel stripes labeled X–Y–Z–W: stripe X corresponds to AB stacking, composed of 15 patches of $\psi_{2,\mathrm{AB}}\left(x\right)$; stripe Z corresponds to BA stacking, composed of 15 patches of $\psi_{2,\mathrm{BA}}\left(x\right)$; stripes Y and W are disordered regions initialized to a constant value $\bar{\psi }$ with small Gaussian noise perturbations. Once time evolution is initiated, the ordered AB and BA domains (X and Z) expand into the disordered regions, forming two transition regions.

**Figure 3:**
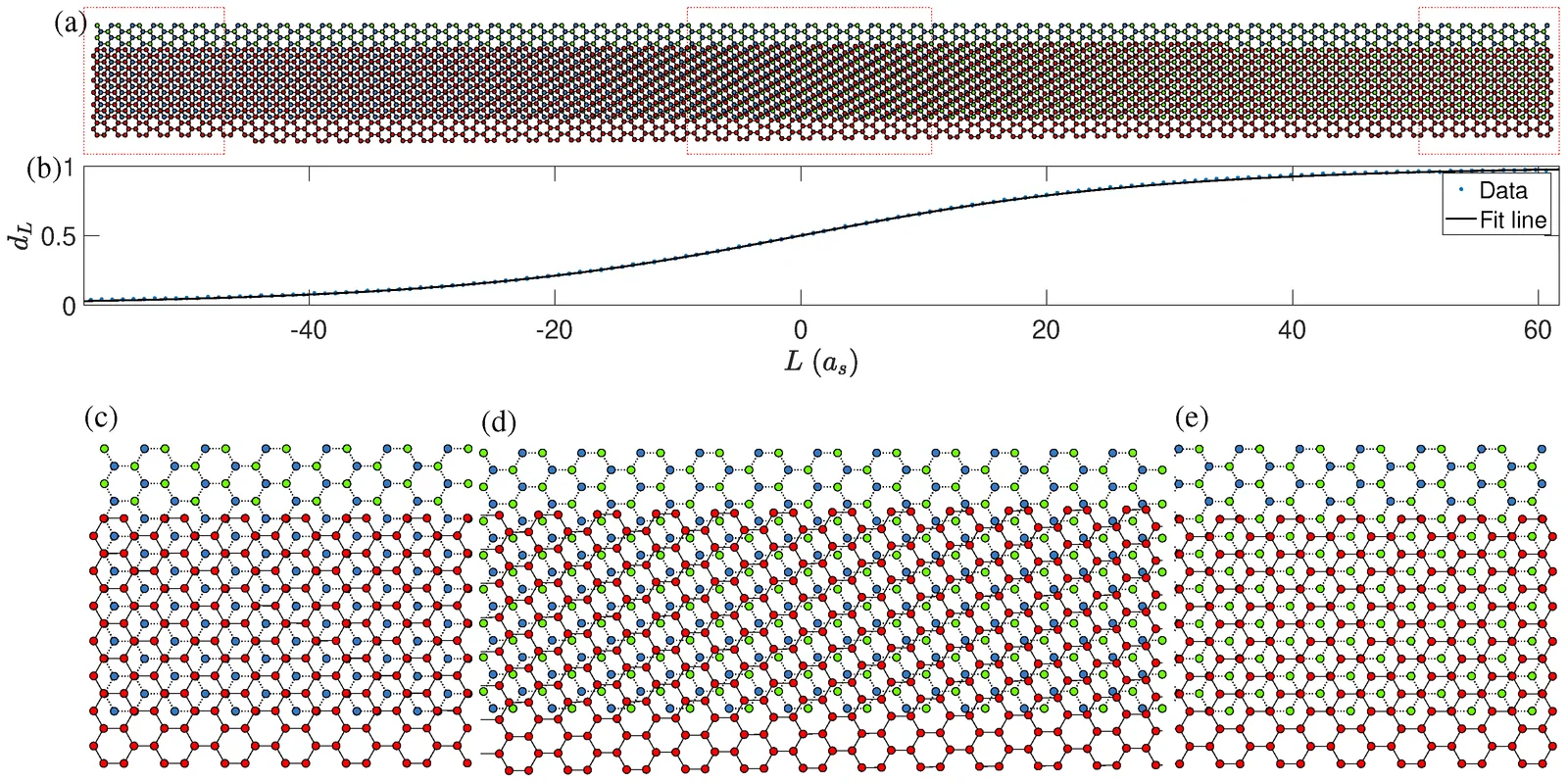
(**a**) Overview of the BA-to-AB transition band, with different types of carbon atoms represented by different colors, following the same convention as in Figure 2; (**b**) plot of $d_{L}$ as a function of *L*, where the horizontal axis is aligned with that of panel (**a**); (**c**) magnified view of the left region in the left red box (uniform BA stacking); (**d**) magnified view of the central region in the middle red box (core of the transition domain boundary); (**e**) magnified view of the right region in the right red box (uniform AB stacking).

Four distinct transition types are observed, classified by the dislocation character angle, defined as the angle between the longitudinal direction of the transition region and the atomic shifting direction: $0^{\circ }$, $30^{\circ }$, $60^{\circ }$, and $90^{\circ }$. For example, the transition regions shown in Figure 3a are vertically oriented, and the dislocation character angle is $30^{\circ }$, as shown in Figure 3c. The strength of the bottom-layer potential is quantified by comparing the measured transition-region widths at each angle with the corresponding atomistic simulation results [27]. Since the transition width *W* at each dislocation character angle reflects the balance between interlayer and intralayer interactions, simultaneous agreement across all angles indicates that the model accurately captures both the magnitude and the anisotropy of the interlayer interaction, thereby serving as a reliable metric for model validation.

To quantify the thickness of the transition region, we define a dimensionless parameter $d_{L}$ as the in-plane distance between the center of an atom of one group on the bottom layer (lighter green) and the center of its nearest carbon atoms on the top layer and, by definition, $d_{L}=1$ for perfect AB stacking and $d_{L}=0$ for perfect BA stacking. Such a parameter quantifies the local lattice distortion induced by the substrate potential $\psi_{1}\left(x\right)$ in the top-layer graphene field $\psi_{2}\left(x\right)$. Following [27], the displacement data are fitted to the function

(13) $$d_{L}=\frac{2}{\pi }\arctan\left(\exp\left(\frac{\pi L}{W}\right)\right),$$

where *W* is a fitting parameter characterizing the thickness of the transition region, and *L* parameterizes the long side of the ribbon domain. The parameter *L* is expressed in terms of $a_{s}=2.461\;Å$, defined as the distance between two adjacent minimum points—that is, the in-plane equilibrium lattice constant of pristine single-layer graphene.

Equation (13), originally proposed to describe strain solitons in bilayer graphene [36], provides an excellent fit to the displacements for both atomistic simulations [27] and XPFC simulations.

Figure 3 illustrates the atomic structure of the domain boundary (transition region) for the BA-to-AB stacking transition in a long bilayer graphene ribbon, along with the quantitative characterization and fitting results of the stacking-order parameter across the transition region. It serves as the core validation figure for calibrating the interlayer interaction strength parameter $\lambda_{\psi }$ in this work. Blue and green atoms correspond to the two sublattices of the bottom graphene layer, while red atoms represent the upper graphene layer.

Figure 3a presents a top-down view of the atomic structure of the full bilayer graphene ribbon, where the horizontal axis corresponds to the longitudinal direction of the ribbon. The entire structure exhibits a continuous smooth transition from BA stacking on the left to AB stacking on the right. Three pink rectangular boxes mark three representative regions—the uniform BA domain on the left, the core of the transition domain boundary in the middle, and the uniform AB domain on the right—which correspond to the three magnified panels below. The dislocation character angle of this transition region is 30°, defined as the angle between the transition region’s orientation and the direction of atomic shift.

In Figure 3b, we plot $d_{L}$ as a function of *L*, and the horizontal axis in Figure 3a corresponds to that in Figure 3b. The data from the numerical simulations nearly coincide with the fitted curve, confirming that the fitting formula in Equation (13) accurately describes the stacking-order parameter profile across the transition region. All the fittings yield $R^{2}>0.9997$ with negligible relative uncertainty.

Figure 3c is the magnified view of the leftmost box in panel (a), showing the BA stacking region before the transition. The top and bottom layers maintain regular BA-type Bernal stacking coordination with negligible lattice distortion. Figure 3d is the magnified view of the middle box in panel (a), showing that the lattice gradually distorts and transitions from BA to AB coordination. Figure 3e, the magnified view of the rightmost box in panel (a), shows the AB stacking region, forming a symmetric stable configuration with the BA domain on the left.

**Figure 4:**
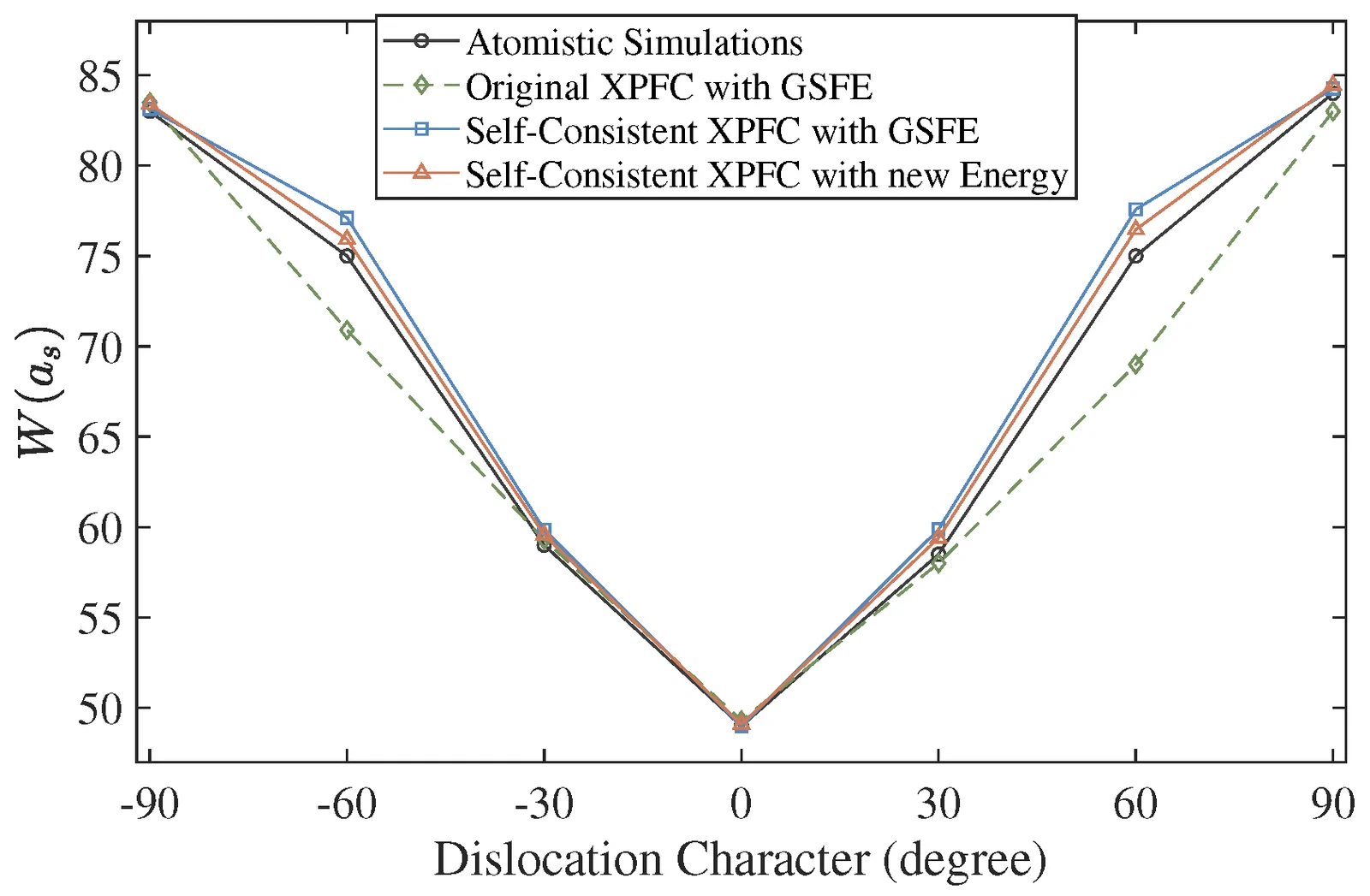
Relation between dislocation direction and transition-region thickness *W*. Here, the dislocation character is defined as the angle between the transition-region direction and the direction along which the atoms shift. The ‘self-consistent XPFC with conventional GSFE’ line corresponds to the calibrated value $\lambda_{F}=2.637\times 10^{5}$, and the ‘self-consistent XPFC with new stacking energy’ line corresponds to $\lambda_{\psi }=1.416\times 10^{3}$.

Mainly considering the steady-state transition widths $W_{0}$ and $W_{\pm 90}$, the optimal value of the interlayer potential strength is determined to be $\lambda_{\psi }=1.416\times 10^{3}$ and $\lambda_{F}=2.637\times 10^{5}$, as shown in Figure 4. Note that, for all the cases here, the bottom layer is fixed. One can easily observe a systematic improvement compared with the atomistic simulations.

### 3.2. Twisted Bilayer Graphene near the First Magic Angle (∼1.1°)

Finally, we apply the self-consistent XPFC model developed above to simulate the twisted bilayer graphene near the first magic angle (∼1.1°), where unconventional superconductivity was discovered in the graphene superlattices [8]. Unlike previous studies where the bottom layer is treated as a rigid fixed substrate [27], both layers are allowed to evolve self-consistently under the dynamic interlayer interaction potential introduced in Section 2, thereby enabling mutual structural relaxation between the two layers. Following Refs. [27,33], we use a supercell method to impose periodic boundary conditions on the twisted layer. The twist angle ϕ between the two layers is calculated by

(14) $$cos\phi =\frac{n^{2}+4nm+m^{2}}{2(n^{2}+nm+m^{2})},$$

where *n* and *m* are the coordinates of the lattice vectors V(m,n) and $V^{\prime }\left(n,m\right)$ with respect to the basis vectors ${\vec{a}}_{1}\left(\sqrt{3}/2,-1/2\right)$ and ${\vec{a}}_{2}\left(\sqrt{3}/2,1/2\right)$.

**Figure 5:**
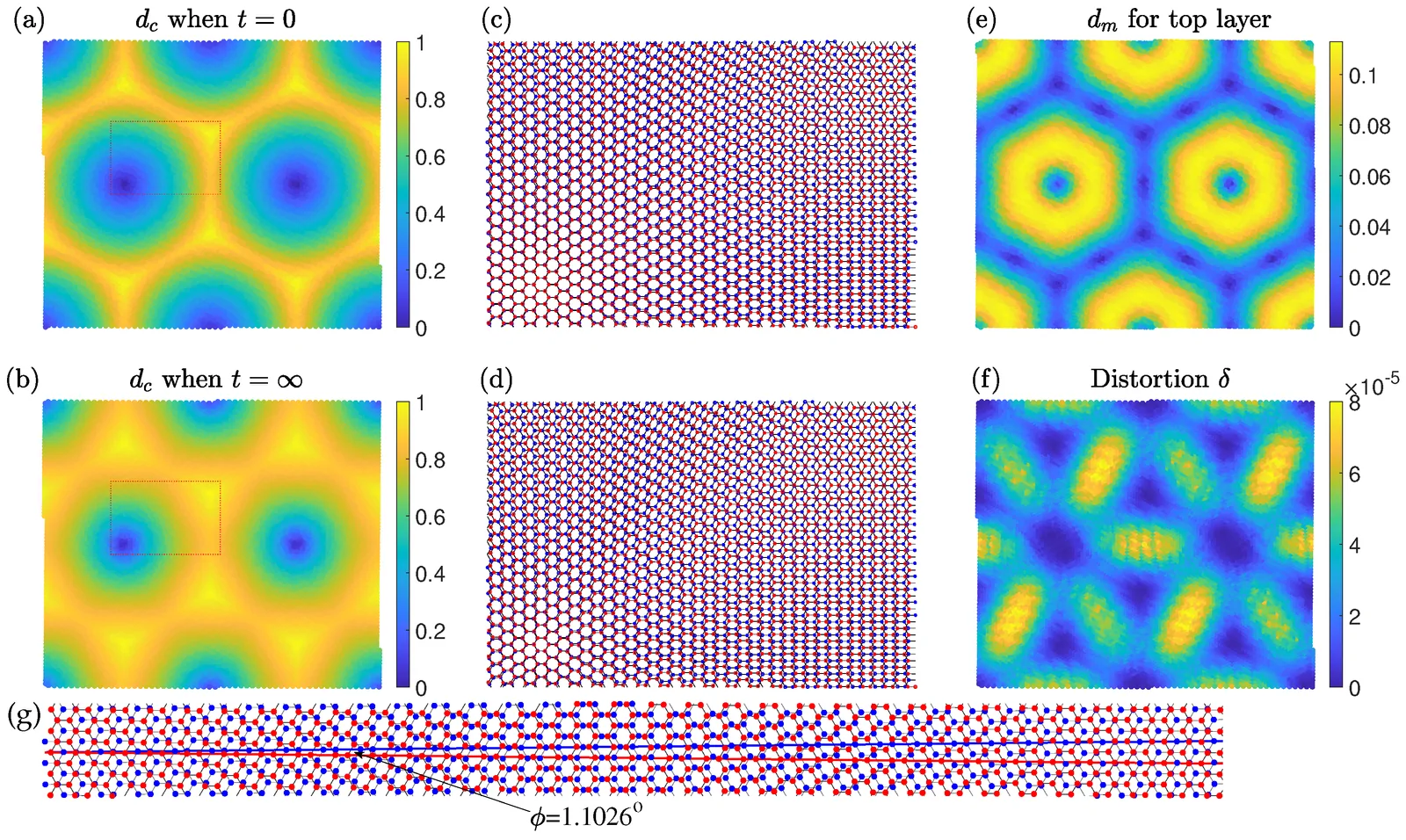
Comparison between the rigid state and the relaxed state. (**a**) Stacking-order parameter $d_{c}$ at rigid state T=0. (**b**) Stacking-order parameter $d_{c}$ at the relaxed state T→∞. (**c**) Real-space atomic configuration at the rigid state for the region in the red box of (a), where the atoms on the bottom layer are denoted by blue dots, and the atoms on the top layer are denoted by red dots. (**d**) Real-space atomic configuration at the relaxed state for the region in the red box of (b). (**e**) In-plane displacement $d_{m}$ of the top layer. (**f**) Local lattice distortion. (**g**) Schematic of 1.1° magic angle.

We set n=59 and m=61, which gives a twist angle $\phi =1.1026^{\circ }$, a good approximation near the first magic angle (∼1.1°) (the combination n=30, m=31 with $\phi =1.085^{\circ }$ is also a reasonable approximation and computationally cheaper), as shown in Figure 5g. We run the simulation until equilibrium is reached. Note that both layers are allowed to evolve dynamically.

Figure 5 illustrates the structural evolution of 1.1° magic-angle twisted bilayer graphene from the rigid state (t=0) to the relaxed state (t→∞). Driven by interlayer van der Waals interactions, the system expands the low-energy AB/BA stacking regions and shrinks high-energy AA stacking regions via in-plane atomic displacement.

In Figure 5a, the parameter $d_{c}$ is defined as the normalized in-plane distance between the center of a hexagonal carbon ring on the bottom layer and the nearest center of a hexagon on the top layer. By definition, $d_{c}=0$ corresponds to AA stacking, while $d_{c}=1$ corresponds to AB or BA (Bernal) stacking. The dark blue circular regions mark the cores of AA stacking, while the yellow regions correspond to configurations close to AB/BA stacking. Together, they form a periodic hexagonal moiré superlattice pattern [33].

Figure 5b shows the stacking-order parameter $d_{c}$ at the relaxed state T→∞. A prominent change is that the dark blue regions shrink while the yellow regions expand substantially. This demonstrates that, driven by interlayer stacking energy, the system evolves toward the lower-energy AB/BA stacking configuration.

Figure 5c,d show the atomic arrangement of bilayer graphene in the dashed-box region of panels (a) and (b). The red circles and dots mark the atoms on the top layer, while the blue circles and dots mark the atoms on the bottom layer. As shown in Figure 5c, the system is initialized with no in-plane atomic relaxation, where every carbon ring takes the form of an ideal regular hexagon shape. Compared with Figure 5d, in-plane displacements of carbon atoms are visible as the atoms relax toward the stable positions of AB/BA stacking, which is consistent with the change in $d_{c}$.

A dimensionless parameter $d_{m}$ is defined as the in-plane displacement of each carbon atom between the initial and equilibrium states

(15) $$d_{m}\left(x_{i}\right)=∥x_{i}\left(\infty \right)-x_{i}\left(0\right)∥,$$

where $x_{i}\left(t\right)=\left(x_{i}\left(t\right),y_{i}\left(t\right)\right)$ is the location of the *i*-th atom at time *t*. The value of $d_{m}$ for the top layer is shown in Figure 5e. A hexagonal periodic pattern emerges, which is consistent with the moiré superlattice. Atomic displacement is negligible at the centers of AA stacking and AB/BA stacking (dark blue regions) and is concentrated in the transition domain boundaries between AA and AB/BA stacking (bright yellow annular regions). Also note that the distribution of $d_{m}$ for the bottom layer is nearly identical to that of the top layer, with barely discernible deviations.

Figure 5f shows the lattice distortion parameter δ that quantifies the deformation of each hexagonal carbon ring compared to an ideal regular hexagonal lattice. It is defined as

(16) $$\delta_{i}=\sum_{i\in K_{k}}{\left(∥x_{i}-y_{k}∥-1\right)}^{2}+{\left(∥x_{i}-x_{i+1}∥-1\right)}^{2},$$

where $y_{k}$ denotes the center of the *k*-th carbon ring, $K_{k}$ is the index set of all the carbon atoms belonging to this ring, and $x_{i+1}$ denotes the position of the atom adjacent to atom *i* in the clockwise direction around the ring. The overall distribution of $\delta_{i}$ presents periodic oblique stripes. The central regions of AA and AB/BA stacking maintain nearly perfect hexagonal lattices with low distortion.

**Figure 6:**
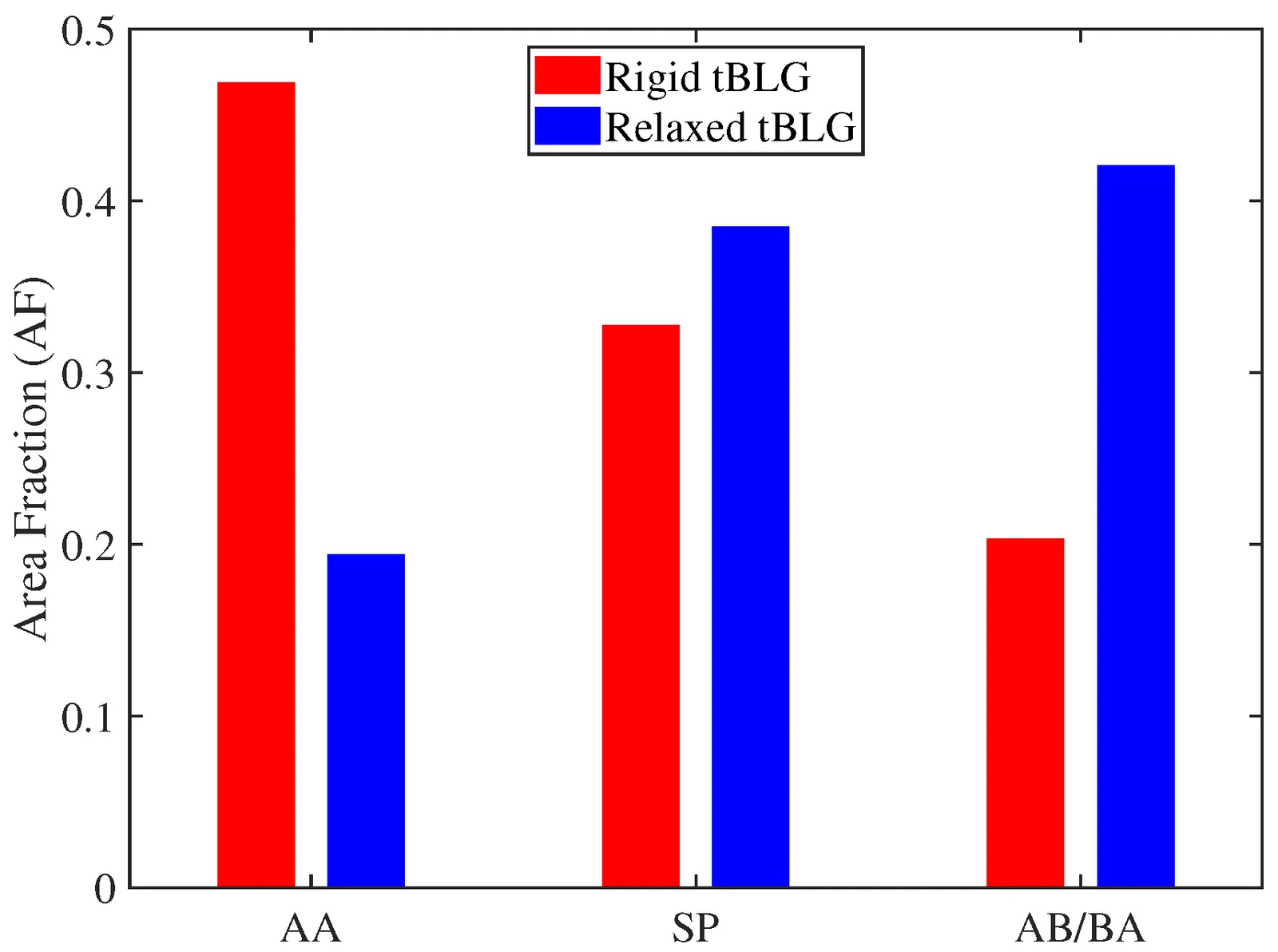
The quantitative area-fraction statistics.

The quantitative area-fraction statistics further confirm the reconstruction effect, as shown in Figure 6. Compared with the rigid structure, the relaxed tBLG shows a pronounced decrease in the area fraction of AA stacking, a remarkable increase in the fraction of AB/BA domains, and a moderate variation in the soliton domain walls (SP transition regions). This reconstruction behavior arises from the balance between interlayer van der Waals attraction and intralayer strain, and it strongly modulates the local structure of tBLG, thereby providing the structural origin of various quantum phenomena in moiré van der Waals heterostructures. See more details in the Supplementary Materials.

## 4. Discussion

This work presents a self-consistent XPFC model for multilayer graphene, achieving improvements in two aspects—the deviation between the equilibrium density field and the first-principles GSFE surface and poor numerical stability—through genetic algorithm optimization of the free-energy parameters. These two improvements share a common origin: as detailed in Supplementary Materials Section S4.4, the optimized parameters confine the density field to a narrow low-amplitude interval, suppressing the high-order nonlinear terms and allowing the linear implicit term to dominate the evolution equation. This separation of scales grants the semi-implicit scheme higher stability, permitting a substantially larger time step and yielding more than ∼100-fold computational speedup, as reported in Supplementary Materials Section S4.4, without sacrificing thermodynamic accuracy. Building on this advancement, we use the self-consistent dynamic interlayer potential to replace the fixed-substrate approximation by allowing both layers to relax mutually rather than treating the bottom layer as rigid.

When applied to the $1.1^{\circ }$ magic-angle tBLG system, the self-consistent model reveals spontaneous structural evolution driven by interlayer vdW interactions, expanding the low-energy AB/BA stacking fraction while shrinking the high-energy AA regions, consistent with the area-fraction statistics. As shown in Supplementary Materials Section S4.6, this reconstruction is accompanied by a reshaping of the local GSFE landscape itself: relative to the rigid lattice, the relaxed configuration exhibits an expanded low-energy regime and narrowed high-energy peaks, while the AB/BA domains coalesce into a percolating network and the SP transition walls are compressed into sharper boundaries.

It is important to note that the current model has a clear scope of application: it is designed specifically for the structural relaxation of bilayer graphene systems and does not account for the material’s electronic properties. The interlayer potential and free-energy parameters are calibrated exclusively for bilayer graphene systems; extending the model to other 2D layered materials, such as hexagonal boron nitride or transition-metal dichalcogenides, will require recalibrating the GSFE surface and interlayer interaction potential parameters to match the specific interlayer vdW characteristics of each material.

Looking ahead, there are several promising directions for further extending this model. First, the interlayer coupling framework established for bilayer systems can be generalized to multilayer graphene and van der Waals heterostructures, such as graphene/hexagonal boron nitride and transition-metal dichalcogenides, thereby establishing a universal XPFC simulation framework for diverse two-dimensional layered materials. Second, additional physical effects, including lattice defects, thermal fluctuations, applied stress, and electric fields, can be incorporated to systematically investigate the dynamics of stacking domain boundaries and the evolution of stability in moiré superlattices under realistic conditions, bringing simulations closer to practical material fabrication and device operation scenarios.

## Data Availability

The original contributions presented in this study are included in the article/Supplementary Materials. Further inquiries can be directed to the corresponding author.

## Supplementary Materials

The following supporting information can be downloaded at: https://www.mdpi.com/article/10.3390/nano16161000/s1, Figure S1: The GSFE energy; Figures S2 and S3: Comparison of MD simulation with experimental results and XPFC simulations; Figure S4: Domain Scaling; Figure S5: Demonstration of moire reconstruction; Figure S6: Evolution of Energy; Table S1: Characteristic Scales in SI Unit System. Table S2: Performance Comparison. Reference [37] is cited in the supplementary materials.

## References

[1] Castro Neto A.H., Guinea F., Peres N.M.R., Novoselov K.S., Geim A.K. (2009). The electronic properties of graphene. Rev. Mod. Phys..

[2] Ohta T., Bostwick A., Seyller T., Horn K., Rotenberg E. (2006). Controlling the electronic structure of bilayer graphene. Science.

[3] Oostinga J.B., Heersche H.B., Liu X., Morpurgo A.F., Vandersypen L.M.K. (2008). Gate-induced insulating state in bilayer graphene devices. Nat. Mater..

[4] Butz B., Dolle C., Niekiel F., Weber K., Waldmann D., Weber H.B., Meyer B., Spiecker E. (2014). Dislocations in bilayer graphene. Nature.

[5] Patel H., Huang L., Kim C.J., Park J., Graham M.W. (2019). Stacking angle-tunable photoluminescence from interlayer exciton states in twisted bilayer graphene. Nat. Commun..

[6] AU Wu J.B., Zhang X., Ijäs M., Han W.P., Qiao X.F., Li X.L., Jiang D.S., Ferrari A.C., Tan P.H. (2014). Resonant Raman spectroscopy of twisted multilayer graphene. Nat. Commun..

[7] Park C.H., Yang L., Son Y.W., Cohen M.L., Louie S.G. (2008). Anisotropic behaviours of massless Dirac fermions in graphene under periodic potentials. Nat. Phys..

[8] Cao Y., Fatemi V., Fang S., Watanabe K., Taniguchi T., Kaxiras E., Jarillo-Herrero P. (2018). Unconventional superconductivity in magic-angle graphene superlattices. Nature.

[9] Yankowitz M., Chen S., Polshyn H., Zhang Y., Watanabe K., Taniguchi T., Graf D., Young A.F., Dean C.R. (2019). Tuning superconductivity in twisted bilayer graphene. Science.

[10] Oh M., Nuckolls K.P., Wong D., Lee R.L., Liu X., Watanabe K., Taniguchi T., Yazdani A. (2021). Evidence for unconventional superconductivity in twisted bilayer graphene. Nature.

[11] Xie Y., Pierce A.T., Park J.M., Parker D.E., Khalaf E., Ledwith P., Cao Y., Lee S.H., Chen S., Forrester P.R. (2021). Fractional Chern insulators in magic-angle twisted bilayer graphene. Nature.

[12] Ghosh A., Chakraborty S., Dutta R., Agarwala A., Watanabe K., Taniguchi T., Banerjee S., Trivedi N., Mukerjee S., Das A. (2025). Thermopower probes of emergent local moments in magic-angle twisted bilayer graphene. Nat. Phys..

[13] Persky E., Parisot L., He M., Cai J., Taniguchi T., Watanabe K., May-Mann J., Xu X., Kapitulnik A. (2026). Optical control of orbital magnetism in magic-angle twisted bilayer graphene. Nat. Phys..

[14] Quinn S., Kong T., Luskin M., Watson A.B. (2025). Higher-order continuum models for twisted bilayer graphene. J. Math. Phys..

[15] Accioly G., Surrente A., Maude D.K., Dumcenco D., Alonso-González P., Vdovin E., Kis A., Panoiu N.C., Dal Conte S., Cerullo G. (2023). Complex Strain Scapes in Reconstructed Transition-Metal Dichalcogenide Moiré Superlattices. ACS Nano.

[16] Li S., Zhang Q., Xing H., Cao Y., Zhang S., Zhang X., Ding B., Li X. (2026). Strain, stress and rotation fields, and energetic features of twisted 2D materials. Natl. Sci. Rev..

[17] Wang Y., Clark D., Das S., Zhu Z., Massatt D., Gavini V., Luskin M., Ortner C. (2025). An atomic cluster expansion potential for twisted multilayer graphene. Mach. Learn. Sci. Technol..

[18] Asle Zaeem M., Thomas S., Kavousi S., Zhang N., Mukhopadhyay T., Mahata A. (2024). Multiscale computational modeling techniques in study and design of 2D materials: Recent advances, challenges, and opportunities. 2D Mater..

[19] Elder K.R., Grant M. (2004). Modeling elastic and plastic deformations in nonequilibrium processing using phase field crystals. Phys. Rev. E.

[20] Elder K.R., Katakowski M., Haataja M., Grant M. (2002). Modeling Elasticity in Crystal Growth. Phys. Rev. Lett..

[21] Meca E., Lowengrub J., Kim H., Mattevi C., Shenoy V.B. (2013). Epitaxial Graphene Growth and Shape Dynamics on Copper: Phase-Field Modeling and Experiments. Nano Lett..

[22] Channe S.S. (2024). Phase-field crystal modeling of graphene/hexagonal boron nitride interfaces. Phys. Chem. Chem. Phys..

[23] Starodumov I., Ankudinov V., Nizovtseva I. (2022). A review of continuous modeling of periodic pattern formation with modified phase-field crystal models. Eur. Phys. J. Spec. Top..

[24] Greenwood M., Rottler J., Provatas N. (2011). Phase-field-crystal methodology for modeling of structural transformations. Phys. Rev. E Stat. Nonlinear Soft Matter Phys..

[25] Seymour M., Provatas N. (2016). Structural phase field crystal approach for modeling graphene and other two-dimensional structures. Phys. Rev. B.

[26] Hirvonen P., Ervasti M.M., Fan Z., Jalalvand M., Seymour M., Vaez Allaei S.M., Provatas N., Harju A., Elder K.R., Ala-Nissila T. (2016). Multiscale modeling of polycrystalline graphene: A comparison of structure and defect energies of realistic samples from phase field crystal models. Phys. Rev. B.

[27] Qiao H., Liu K. (2025). Phase-Field Crystal Method for Bilayer Graphene. Nanomaterials.

[28] Zhou S., Han J., Dai S., Sun J., Srolovitz D.J. (2015). Van der Waals bilayer energetics: Generalized stacking-fault energy of graphene, boron nitride, and graphene/boron nitride bilayers. Phys. Rev. B.

[29] Van Swygenhoven H., Derlet P.M., Frøseth A.G. (2004). Stacking fault energies and slip in nanocrystalline metals. Nat. Mater..

[30] Feng L., Mills M.J., Wang Y. (2021). Generalized stacking fault energy surface mismatch and dislocation transformation. npj Comput. Mater..

[31] Holland J.H. (1992). Genetic Algorithms. Sci. Am..

[32] Fox C., Mao Y., Zhang X., Wang Y., Xiao J. (2024). Stacking Order Engineering of Two-Dimensional Materials and Device Applications. Chem. Rev..

[33] Trambly de Laissardière G., Mayou D., Magaud L. (2010). Localization of Dirac Electrons in Rotated Graphene Bilayers. Nano Lett..

[34] Nam N.N.T., Koshino M. (2017). Lattice relaxation and energy band modulation in twisted bilayer graphene. Phys. Rev. B.

[35] Dey A., Chowdhury S.A., Peña T., Singh S., Wu S.M., Askari H. (2023). An Atomistic Insight into Moiré Reconstruction in Twisted Bilayer Graphene beyond the Magic Angle. ACS Appl. Eng. Mater..

[36] Alden J.S., Tsen A.W., Huang P.Y., Robert H., Lola B., Jiwoong P., Muller D.A., Mceuen P.L. (2013). Strain solitons and topological defects in bilayer graphene. Proc. Natl. Acad. Sci. USA.

[37] Mostaani E., Drummond N.D., Fal’ko V.I. (2015). Quantum Monte Carlo calculation of the binding energy of bilayer graphene. Phys. Rev. Lett..

