# Supplementary material for: A Self-Consistent Phase Field Crystal Method for Twisted Bilayer Graphene
Source: Nanomaterials (Basel). 2026 Aug 14;16(16):1000. doi: 10.3390/nano16161000 (PMC13515764; doi:10.3390/nano16161000)
Supplement: Supplementary file 1 [file nanomaterials-16-01000-s001.zip › nanomaterials-4449929-supplementary.pdf]

Supporting Information

Pingqia Wang, Kai Liu

S1 GSFE-based Potential

This formulation uses a precomputed fixed potential field derived from the first-principles GSFE surface of a rigid graphene substrate. It is defined as $V_{BL}(\mathbf{x}) = \frac{1}{\lambda_F} F_{\text{GSFE}}(\mathbf{x})$, where $F_{\text{GSFE}}(\mathbf{x})$ is the analytical GSFE function fitted to first-principles data, and λ_F is a dimensionless calibration parameter that absorbs the dimensional conversion between the GSFE energy scale and the dimensionless free energy framework of the XPFC model. λ_F represents the strength of the stacking energy and will be determined later. The $F_{\text{GSFE}}(\mathbf{x})$ is given as

$$
\begin{aligned}
F_{\text{GSFE}}(\mathbf{x}) = c_0 &+ c_1 \left[\cos \frac{2\pi}{a_s} \left(x + \frac{y}{\sqrt{3}} \right) + \cos \frac{2\pi}{a_s} \left(x - \frac{y}{\sqrt{3}} \right) + \cos \frac{4\pi y}{\sqrt{3} a_s} \right] \\
&+ c_2 \left[\cos \frac{2\pi}{a_s} \left(x + \sqrt{3} y \right) + \cos \frac{2\pi}{a_s} \left(x - \sqrt{3} y \right) + \cos \frac{4\pi x}{a_s} \right] \\
&+ c_3 \left[\cos \frac{2\pi}{a_s} \left(2x + \frac{2y}{\sqrt{3}} \right) + \cos \frac{2\pi}{a_s} \left(2x - \frac{2y}{\sqrt{3}} \right) + \cos \frac{8\pi y}{\sqrt{3} a_s} \right] \\
&+ c_4 \left[\sin \frac{2\pi}{a_s} \left(x - \frac{y}{\sqrt{3}} \right) - \sin \frac{2\pi}{a_s} \left(x + \frac{y}{\sqrt{3}} \right) + \sin \frac{4\pi y}{\sqrt{3} a_s} \right] \\
&+ c_5 \left[\sin \frac{2\pi}{a_s} \left(2x - \frac{2y}{\sqrt{3}} \right) - \sin \frac{2\pi}{a_s} \left(2x + \frac{2y}{\sqrt{3}} \right) + \sin \frac{8\pi y}{\sqrt{3} a_s} \right],
\end{aligned} \tag{S1}
$$

where a_s is the distance between two nearby minimum points, i.e., two nearby holes on the bottom layer of graphene. $c_0 = 21.336$, $c_1 = 12.254$, $c_2 = -1.128$, $c_3 = -0.286$, $c_4 = \sqrt{3} c_1$, and $c_5 = -\sqrt{3} c_3$ [4]. The details of the F_{GSFE} is shown in Figure S1.

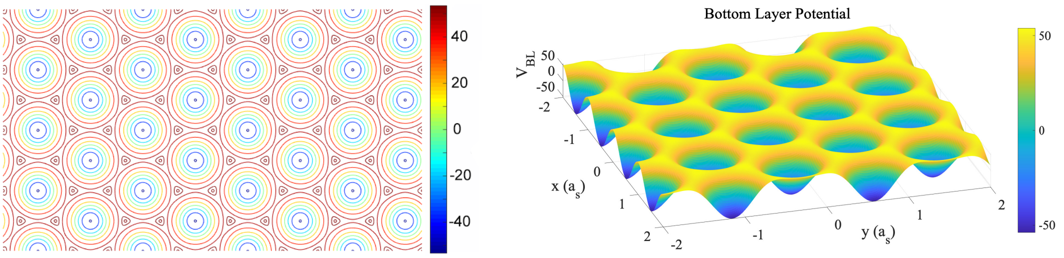

Figure S1: The GSFE energy when $\lambda_F = 1$. The maximum and the minimum points are the location of carbon atoms and holes on the bottom layer correspondingly.

S2 Atomistic Modeling

Alden et al. measured boundary widths in bilayer graphene by STEM [1], extracting them from simulated images based on atomic displacements given by

$$d_L = \frac{2}{\pi} \arctan \left[\exp \left(\frac{\pi L}{W} \right) \right] , \tag{S2}$$

where d_L is the fractional displacement. Their primary samples consisted of four freestanding graphene layers with the inner two layers near Bernal stacking; the outer layers were far from optimal stacking angles, only weakly perturbing the in-plane positions of the Bernal-stacked pair while keeping them flat. Our most relevant atomistic model therefore keeps the bottom layer flat but allows in-plane relaxation, and the boundary widths from these simulations agree with Alden et al.'s results.

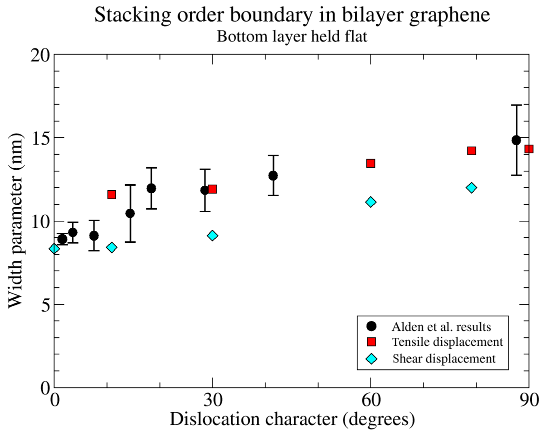

Figure S2: Comparison of the widths of the boundaries in our MD simulation to those in Figure 3H of reference [1]. The horizontal axis is the angle between the line of the boundary and the burgers vector of the boundary. The vertical axis is the parameter w in Equation (S2).

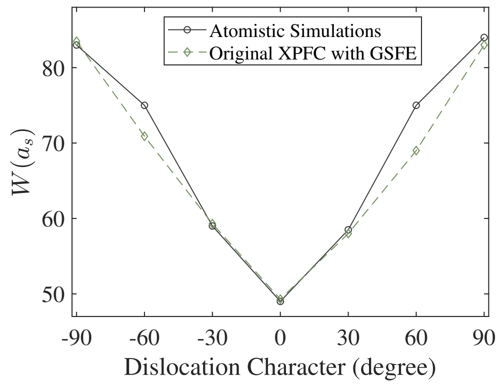

Figure S3: The relation between the dislocation direction and W when $\lambda = 7500$. The results presented in the figure are calculated with respect to the original XPFC model, i.e. the coefficients are the same as those in [3].

In Figure S2 we compare our simulated widths with the experimental values extracted from Alden et al.'s Figure S2. The widths in that figure are reported as the full width at half-maximum (FWHM) of the STEM intensity profile; we convert them to the parameter W in Equation (S2) using the relation given in the Supplemental Material of Ref. [1] (data extracted with Graph Grabber 2).

Equation (S2) provides an excellent fit to the displacements in both atomistic and XPFC simulations. For the flat bottom simulations we have examined the displacement parallel to the boundary (shear displacement) and normal to the boundary (tensile displacement) separately for the mixed dislocations. We find that the Eq. (S2) fits the separate displacements very well, but that the widths W differ between the shear and tensile components. That equation also fits the component of the displacement parallel to the burgers vector, which is what we have fitted for the fixed bottom simulations for comparison with the XPFC, as shown in Figure S3.

S3 Scaling and Non-Dimensionalization Process

To ensure the full reproducibility of the modified XPFC model and eliminate all ambiguities related to physical units, parameter mapping, and numerical implementation logic, all physical quantities in this work are non-dimensionalized against intrinsic characteristic scales of bilayer graphene, following the International System of Units (SI). This section systematically defines all reference scales, dimensional physical quantities, conversion formulas, and corresponding relationships between dimensionless model parameters and practical physical values.

S3.1 Basic Principles of Non-Dimensionalization

For the two-dimensional twisted bilayer graphene (tBLG) system, we select 3 independent fundamental reference scales:

a. Length scale: Determined by the intrinsic lattice constant of graphene, used to normalize spatial coordinates and grid size;

b. Energy scale: Determined by the intrinsic interlayer binding energy of bilayer graphene, used to normalize all free energy terms;

c. Time scale: Determined by the atomic mobility of graphene, used to normalize the dynamic evolution time.

These reference scales are derived from first-principles calculations or experimental characterization data of pristine graphene, ensuring the physical rationality of the dimensionless framework.

S3.2 Fundamental Reference Scales

All physical quantities are nondimensionalized by their respective characteristic scales, and the values of the dimensionless physical quantities are used throughout the numerical implementation. The core reference scales are listed in Table S1.

Table S1: SI Unit System

Reference Scalec	Symbol	SI Unit Value
Length Scale	Δx	4.807×10^{-12} m
Energy Scale	ε_0	1.035×10^{-19} J
Time Scale	τ_0	1.60×10^{-14} s
Mobility Scale	M_{phys}	3.2×10^{-13} m$^2 \cdot$ s/kg

S3.3 Conversion Formulas for Core Physical Quantities

S3.3.1 Length and Spatial Discretization

In the numerical simulations, the dimensionless grid spacing is uniformly set to unity. This choice constitutes the essential resolution requirement for accurately resolving the hexagonal lattice structure of graphene. Taking the in-plane equilibrium lattice constant of pristine monolayer graphene as $a_0 = 2.461 \times 10^{-10}$ m, the physical spatial resolution is determined via the geometric relation

$$\Delta x = \frac{10a_s}{256 \times 2} = 4.807 \times 10^{-12}\mathrm{m}.$$

The basic rectangular patch is discretized with 256 nodes per side. The dimensionless in-plane spatial coordinate vector $\mathbf{x}^*$ is subsequently defined by normalizing the dimensional coordinate $\mathbf{x}$ with respect to this physical resolution:

$$\mathbf{x}^* = \frac{\mathbf{x}}{\Delta x} \tag{S3}$$

For the simulation of the $1.1°$ magic-angle tBLG moiré superlattice, the dimensionless computational domain comprises 4096×4096 grid points, corresponding to a physical domain size of $L = 4096 \times \Delta x \times l_{\text{scale}} \approx 511\,\text{nm}$, which is sufficiently large to eliminate the artificial finite-size effects arising from periodic boundary conditions. Here the coefficient $l_{\text{scale}} = 2.598$ ensures compatibility with the periodic boundary conditions of the rectangular computational domain.

S3.3.2 Energy Non-Dimensionalization

The non-dimensionalization of energy follows a standard proportional relationship:

$$E^* = \frac{E_{\text{phys}}}{\varepsilon_0}$$

where ε_0 is the characteristic energy that defines the dominant energy scale of the XPFC model and corresponds to the characteristic magnitude of short-range interatomic repulsion and atomic cohesive energy.

The widely recognized high-precision results from quantum Monte Carlo (QMC) calculations are adopted as the physical calibration benchmark. The per-atom energy difference between AA and AB stacking in bilayer graphene is given by

$$\Delta E_{\text{phys, atom}} = 17.7 - 11.5 = 6.2 \text{ meV/atom} = 9.93 \times 10^{-22} \text{ J/atom}.$$

This result is on the same order of magnitude as the generalized stacking fault energy (GSFE) values computed via the adiabatic connection fluctuation-dissipation theorem with the random phase approximation (ACFDT-RPA) method, and thus serves as a reliable physical anchor for energy non-dimensionalization [2, 4].

The value of $E^* = 9.6 \times 10^{-3}$ per atom obtained from the simulation represents the dimensionless energy difference per atom: it is calculated as the total dimensionless free energy difference between the AA and AB stacking configurations, divided by the total number of carbon atoms in the system. This quantity is a purely dimensionless value, representing a relative result under the intrinsic energy scale of the XPFC model.

By substituting the known physical stacking energy difference and the simulated dimensionless energy difference into the above equation, the characteristic energy reads:

$$\varepsilon_0 = \frac{\Delta E_{\text{phys, atom}}}{\Delta E^*_{\text{atom}}} = \frac{6.2 \text{ meV/atom}}{0.0096\text{/atom}} \approx 646 \text{ meV} \approx 0.646 \text{ eV}.$$

Converted to the International System of Units (SI):

$$\varepsilon_0 \approx 0.646 \times 1.602 \times 10^{-19}\,\mathrm{J} \approx 1.035 \times 10^{-19}\,\mathrm{J}\,.$$

S3.3.3 Time Evolution and Mobility Coefficient

The time non-dimensionalization of the XPFC model is established based on pre-calibrated length and energy scales. The XPFC dynamics follows a conservative evolution equation. The characteristic time τ_0, representing the physical duration corresponding to one dimensionless time unit, satisfies the relation $\tau_0 = \Delta x^4/(M_{\text{phys}} \cdot \varepsilon_0)$, where M_{phys} denotes the effective physical mobility of the density field.

To determine M_{phys}, we calibrate the model against the typical room-temperature migration velocity of van der Waals domain walls in bilayer graphene ($v_{\text{phys}} \approx 3 \times 10^2$ m/s). In dimensionless simulations, the steady-state domain wall velocity takes a representative value of $v^* \approx 1$ lattice per dimensionless time unit, consistent with numerical stability constraints. Substituting into the velocity scaling relation $v_{\text{phys}} = v^* \cdot \Delta x/\tau_0$ yields the calibrated mobility.The resulting effective physical mobility is $M_{\text{phys}} \approx 3.2 \times 10^{-13}$ $\mathrm{m}^2 \cdot$ s/kg, with a corresponding characteristic time $\tau_0 \approx 1.60 \times 10^{-14}$ s. This time scale aligns with the atomic-resolution diffusion relaxation regime, ensuring physically interpretable dynamic evolution in XPFC simulations.

Note that the above-derived physical mobility coefficient and characteristic time scale serve solely as a theoretical reference for establishing the dimensional mapping between the XPFC model and real bilayer graphene systems. In practical numerical implementations, the dimensionless mobility coefficient is uniformly set to one to simplify the evolution equation, and the selection of the time step size is primarily determined by the numerical stability constraint of the adopted semi-implicit Fourier spectral scheme, rather than being strictly bounded by the absolute value of the physical mobility. To maximize computational efficiency, the time step is set to the largest admissible value under the current parameter configuration, provided that numerical stability is maintained and the temporal resolution of structural evolution remains sufficient.

S4 Full Discretization, Linear Stability Analysis, and Numerical Verification of the Semi-Implicit Scheme

This section presents the complete discretization derivation, linear stability analysis, and numerical verification data for the semi-implicit scheme, thereby providing a reproducible demonstration of its stability advantage over conventional explicit and implicit approaches.

S4.1 FFT method

In the PFC model, the density n represents a conserved order parameter. As a result, its evolution is described by

$$\frac{\partial \psi}{\partial t} = M_\psi \nabla^2 \left(\frac{\delta F_{\text{total}}}{\delta \psi} \right), \tag{S4}$$

where M_ψ an effective mobility that sets the scale of the diffusional dynamics of ψ, and we set $M_\psi = 1$ for convenience.

We use a discrete Fourier transform (DFT) method to solve Eq. (S4). For two points correlation structure $F_{\text{ex},2}$, we can have [3]

$$\frac{\partial F_{\text{ex},2}}{\partial \psi} = -\int C_2(\mathbf{x} - \mathbf{x}')\psi(\mathbf{x}')d\mathbf{x}' \equiv -C_2 * \psi, \tag{S5}$$

where $*$ is a convolution in the domain, and in the reciprocal space, that

$$\hat{C}_2(\mathbf{k}) = -2R\frac{J_1(r_0 k)}{r_0 k}, \quad k = \sqrt{k_1^2 + k_2^2}, \tag{S6}$$

where J_m are the Bessel functions of the first kind, and k_1, k_2 are the modes in the 2D reciprocal space. The three points correlation structure $C_3(\mathbf{r} - \mathbf{r}', \mathbf{r} - \mathbf{r}'')$ is separated to

$$C_3(\mathbf{r} - \mathbf{r}', \mathbf{r} - \mathbf{r}'') = \sum_i C_s^{(i)}(\mathbf{r} - \mathbf{r}')C_s^{(i)}(\mathbf{r} - \mathbf{r}''). \tag{S7}$$

The $F_{ex,3}$ is then computed as [3]

$$\frac{\partial F_{ex,3}}{\partial \psi} = -\frac{1}{3}\left([C_s^{(i)} * \psi]^2 + 2(-1)^m C_s^{(i)} * [\psi \cdot (C_s^{(i)} * \psi)]\right), \tag{S8}$$

where

$$\hat{C}_s^{(1)}(k, \theta_k) = X i^m \cos(m\theta_k) J_m(k a_0), \tag{S9}$$

$$\hat{C}_s^{(2)}(k, \theta_k) = X i^m \sin(m\theta_k) J_m(k a_0), \tag{S10}$$

and

$$k = \sqrt{k_1^2 + k_2^2}, \quad \theta_k = \arctan\left(\frac{k_2}{k_1}\right). \tag{S11}$$

S4.1.1 Scaling the domain to incorporate the GSFE.

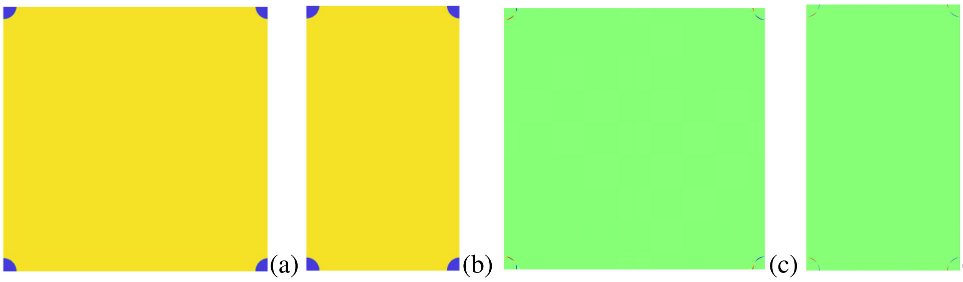

(a) (b) (c) (d)

Figure S4: (a) C_2 for square domain, (b) C_2 for rectangular domain $L \times \sqrt{3}L$. (c) $C_s^{(1)}$ for square domain, (d) $C_s^{(1)}$ for rectangular domain $L \times \sqrt{3}L$.

In order to incorporate the F_{GSFE} into the model, we need to make sure that $F_{\text{GSFE}}(\mathbf{x})$ is also periodic in the domain, i.e., the aspect ratio of the rectangular domain has to be $n_1 : \sqrt{3}n_2$, where n_1 and n_2 are positive integers. For rectangular domain $L \times \sqrt{3}L$, by setting

$$k = \sqrt{k_1^2 + k_2^2 \times 3}, \quad \theta_k = \arctan\left(\frac{k_2 \times \sqrt{3}}{k_1}\right), \tag{S12}$$

C_2 and $C_s^{(i)}$ functions are then of circular shapes, as shown in Fig. S4.

S4.1.2 Discretization of the Laplacian operator

Let $\hat{\psi}_i(k,t)$ denote the Fourier transform of the real-space density field $\psi_i(\mathbf{x},t)$, where $k=(k_x,k_y)$ is the dimensionless wave vector. For the dimensionless computational setting corresponding to $\Delta x=\Delta y=1$, the multiplier simplifies to $-\hat{\nabla}^2(k)=4-2\cos k_x-2\cos k_y$, converting the spatial differential operator into an algebraic operation that is easy to handle numerically.

S4.2 Splitting of the Free Energy Functional Derivative

To ensure the efficacy o f t he s emi-implicit s cheme, t he f unctional d erivative o f t he t otal f ree energy is decomposed into a linear implicit term—which dominates at high wave numbers and thus governs numerical stiffness—and a nonlinear explicit term whose magnitude remains small within the optimized parameter regime:

$$\frac{\delta F_{\text{total},i}}{\delta \psi_i}=\underbrace{\psi_i}_{\text{Linear implicit term}}+\underbrace{\left(-\eta\frac{\psi_i^2}{2}+\chi\frac{\psi_i^3}{3}+f_2\frac{\delta F_{\text{ex},2}}{\delta\psi_i}+f_3\frac{\delta F_{\text{ex},3}}{\delta\psi_i}+\frac{\delta F_{\text{IL}}}{\delta\psi_i}\right)}_{\text{Nonlinear explicit term}} \tag{S13}$$

The linear implicit term arises exclusively from the quadratic contribution of the ideal free energy F_{id}; all nonlinear contributions are consolidated into the explicit component so as to minimize computational complexity.

S4.3 Time Discretization in Fourier Space

Using first-order backward Euler for the implicit linear term and first-order forward Euler for the nonlinear explicit term, the semi-implicit iterative equation in Fourier space is derived as follows:

$$\frac{\hat{\psi}_i^{n+1}-\hat{\psi}_i^n}{\Delta t}=-M_{\psi_i}\cdot\hat{\nabla}^2(k)\cdot\left[\hat{\psi}_i^{n+1}+\left(\widehat{\frac{\delta F_{\text{total},i}}{\delta\psi_i}}\right)^n-\hat{\psi}_i^n\right], \tag{S14}$$

where $\hat{\psi}_i^n$ is the Fourier-space density field at the n-th time step, Δt is the dimensionless time step, k, and $\left(\widehat{\frac{\delta F_{\text{total},i}}{\delta\psi_i}}\right)^n$ is the Fourier transform of the total free energy derivative calculated explicitly from the real-space field at the n-th step. Rearranging the terms to solve for the density field at the next time step $\hat{\psi}_i^{n+1}$, we obtain the final update rule:

$$\hat{\psi}_i^{n+1}=\frac{\hat{\psi}_i^n}{1+M_{\psi_i}\hat{\nabla}^2(k)\Delta t}-\frac{M_{\psi_i}\hat{\nabla}^2(k)\Delta t}{1+M_{\psi_i}\hat{\nabla}^2(k)\Delta t}\left[\left(\widehat{\frac{\delta F_{\text{total},i}}{\delta\psi_i}}\right)^n-\hat{\psi}_i^n\right] \tag{S15}$$

Note that $\left[1+M_{\psi_i}\hat{\nabla}^2(k)\Delta t\right]\geq 1$. This formulation is computationally efficient: each iteration requires only two FFT/IFFT operations and scalar algebraic operations for each wave vector k, making it fully compatible with GPU acceleration.

S4.4 Stability Analysis and the Parameter Optimization Mechanism

The stability of the semi-implicit scheme hinges on the relative magnitude of the linear implicit term versus the nonlinear explicit term—a balance that is precisely regulated through genetic-algorithm optimization of the XPFC model parameters.

S4.4.1 Stability Breakdown Under the Original Parameter Set

In the original XPFC model configuration, the density field varies over a wide range: $\psi_i \in [-3.5, 3.3]$. In this regime, the nonlinear quadratic (ψ_i^2) and cubic (ψ_i^3) terms dominate in magnitude over the linear term ψ_i. Classical stability analysis reveals that the explicit treatment of these large-amplitude nonlinear terms gives rise to unbounded error amplification for high-frequency Fourier modes whenever the time step exceeds the stringent explicit stability limit.

Numerical tests confirm that for the original parameter set, the maximum stable dimensionless time step is restricted to $\Delta t_{\text{max, original}} = 3.7 \times 10^{-5}$. Larger time step causes the density field to diverge within dozens of iteration steps, making long-time relaxation simulations computationally infeasible.

S4.4.2 Unconditional Stability Under the Optimized Parameter Set

Genetic-algorithm optimization calibrates the free-energy coefficients so as to confine the density field to a narrow, low-amplitude interval: $\psi_i \in [-0.16, 0.42]$. In this range, the nonlinear terms are significantly suppressed: the maximum value of the quadratic term is 0.09, and the maximum value of the cubic term is 0.05—both are smaller in magnitude than the linear term ψ_i.

This separation of scales ensures that the linear implicit term dominates the evolution equation. The implicit treatment of the linear term eliminates the strict stability constraint associated with high-frequency modes. And the time step size is no longer limited by stability requirements, but only by the temporal accuracy needed to resolve moiré superlattice relaxation.

S4.4.3 Numerical Verification of Stability and Efficiency

The dynamics are solved by a pseudo-spectral method based on the fast Fourier transform (FFT). By converting convolutions in the free-energy functional to element-wise multiplications in Fourier space, the computational cost is reduced from $O(N^2)$ to $O(N \log N)$. To meet the demanding cost of large-domain tBLG simulations, we reimplemented the solver in CUDA C/C++. We conducted a systematic performance benchmarking test to quantitatively verify the computational efficiency improvement of the modified solver.

The stopping criterion for the iterative relaxation is defined as a relative change in the total system of less than 10^{-8} per 1000 iteration steps.

Stability Performance: The original parameter group showed obvious numerical instability: increasing the time step to 4×10^{-5} would cause the maximum absolute value of the density field to exceed 10^4 within 100 steps. In contrast, the optimized parameter group remained completely stable at $\Delta t = 0.1$; no high-frequency error amplification was observed throughout the whole process.

Computational Efficiency Gain: The original parameter group required 3.8×10^6 iteration steps to reach the convergence criterion, with a total wall-clock time of approximately 53 hours. The optimized parameter group required only 7×10^4 iteration steps to reach the same convergence criterion, with a total wall-clock time of approximately 34 minutes. The overall computational efficiency was improved by a factor of 10^2.

Accuracy Consistency: The equilibrium moiré superlattice structure and interlayer relaxation energy calculated by the two schemes were consistent: the relative error in the total relaxation energy was less than 0.02%, and the maximum deviation of the local stacking position was less than 0.01 Å. This confirms that the large time step in the optimized scheme does not sacrifice calculation accuracy.

S4.5 GPU Acceleration

The test configuration is as follows:

GPU Solver Test Bed: NVIDIA Geforce 3090 24GB GPU; CUDA Toolkit 11.0.

CPU Solver Test Bed: AMD Ryzen 7 5700X desktop processor 8 cores; MATLAB 2021a; original pseudo-spectral solver with the same numerical precision and physical parameters.

Benchmark Case: 1.1° magic-angle tBLG, computational domain size 4096×4096 grid points, 10^5 steps, periodic boundary conditions applied in both in-plane directions.

The CPU version required 41.7 h, whereas the GPU version finished in 21 min, yielding a 115× speedup. The acceleration is mainly attributed to alleviating the FFT bottleneck. For the GPU-accelerated solver, the dominant bottleneck is memory bandwidth—double-precision floating-point arithmetic capacity remains abundant. In contrast, the CPU-based solver is fundamentally compute-bound, with its performance limited by the double-precision FLOPs of the CPU.

Table S2: Performance Comparison

Size of Domain	256×256	1024×1024	256×16384	4096×4096	8192×8192
Time per Step(CPU)	0.01s	0.2s	1.9s	13s	61s
Time per Step(GPU)	0.0002s	0.004s	0.02s	0.1s	0.5s

The largerst GPU memory cost for 8192×8192 resolution is 10Gb for all the variables together.

S5 Demonstration of Moiré Lattice Reconstruction in Twisted Bilayer Graphene

Figure S5 illustrates the structural reconstruction of the moiré superlattice in twisted bilayer graphene (tBLG) by comparing rigid and atomically relaxed configurations, alongside their corresponding gen-eralized stacking fault energy (GSFE) distributions and quantitative area fractions of distinct stacking domains.

In the rigid tBLG case, where the two graphene layers retain their ideal crystalline lattices without in-plane structural relaxation, the stacking orders form a well-defined periodic moiré pattern. AA-stacked regions, with GSFE$>$ 13.2 meV/atom, occupy the dominant area fraction as large periodic domains, while AB/BA-stacked regions, with GSFE$<$ 12.15 meV/atom appear as discrete triangular zones embedded in a continuous network of soliton domain walls (SP transition regions). The corresponding GSFE landscape exhibits a uniform periodic energy profile, with energy maxima located at AA stacking sites and minima at AB/BA sites, which is purely governed by the geometric interlayer registry.

Driven by the minimization of interlayer van der Waals interaction energy, significant moiré reconstruction takes place after atomic relaxation. The thermodynamically favorable AB/BA stacking domains expand substantially, whereas the high-energy AA-stacked domains shrink drastically into compact rounded regions. Meanwhile, the SP transition domain walls also shift to new regions separating distinct stacking phases. In accordance with the structural evolution, the relaxed GSFE map displays an expanded low-energy regime and narrowed high-energy peaks, indicating an overall reduction in the system's interlayer energy.

S6 Evolution of Free Energy

Figure S6 presents the temporal evolution of per-atom free energy and its individual components during the consolidation process of the top layer graphene on a fixed substrate, plotted as a function of dimensionless time. Note that all individual energy components are referenced to zero at the initial state to allow for

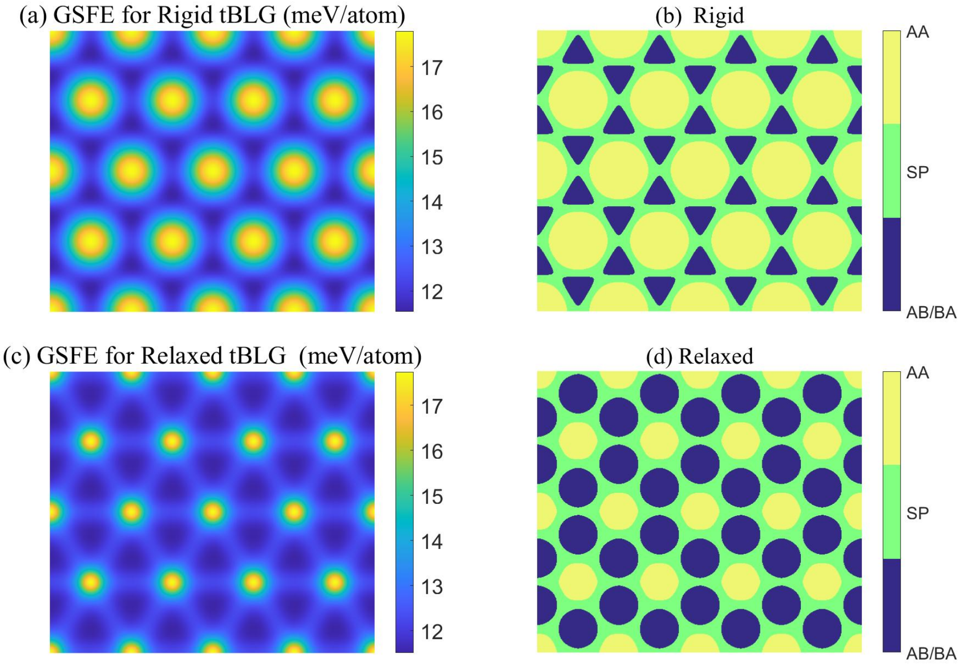

Figure S5: Demonstration of moire reconstruction. (a) the GSFE for the rigid tBLG systems; (b) Stacking contour plot for the rigid tBLG systems; (c) the GSFE for the relaxed tBLG systems; (d) Stacking contour plot for the relaxed tBLG systems.

a consistent and direct comparison of their temporal evolution throughout the entire structural relaxation process.

The total free energy F_{total} (solid black line) starts from the reference zero-energy initial state and decreases monotonically throughout the relaxation process. It undergoes a significant drop in the early stage ($t^* < 2$) and gradually converges to a steady-state value of approximately -2.8eV per atom, indicating that the system spontaneously evolves toward a thermodynamically stable configuration and completes the evolution.

The energy evolution is governed by the competition among multiple contributions. The dominant stabilizing term is $F_{\text{ex,2}}$ (blue dashed line), which declines rapidly to around -4.1 eV at equilibrium and accounts for the majority of cohesive energy gain from strong in-plane covalent bonding, serving as the primary driving force for structural stabilization. In contrast, F_{id} (red dashed line) rises and saturates at approximately 2.4 eV. The $F_{\text{ex,3}}$ term (green dotted line) provides a secondary stabilizing contribution, reaching about -1.0 eV at equilibrium, which originates from next-nearest-neighbor interactions and correlation effects that further lower the system energy. The interlayer interaction term F_{IL} (magenta solid line) remains nearly constant at around 0 eV over the entire process, verifying that the variation of interlayer van der Waals energy is small compared with the in-plane covalent energy scale, consistent with the orders-of-magnitude difference between the two types of interactions.

Overall, the two-stage energy profile reflects the kinetics of bilayer graphene: the fast energy variation in the initial regime corresponds to rapid atomic rearrangement and the establishment of the stable bonding network, while the energy plateau in the late stage marks that the system has reached its minimum-energy equilibrium state.

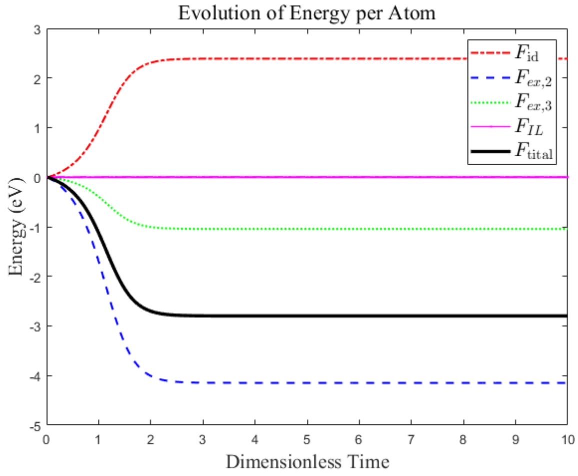

Figure S6: Evolution of the energy.

References

[1] Jonathan S Alden, Adam W Tsen, Pinshane Y Huang, Hovden Robert, Brown Lola, Park Jiwoong, David A Muller, and Paul L Mceuen. Strain solitons and topological defects in bilayer graphene. *Proc. Natl. Acad. Sci.*, 110(28):11256–11260, 2013.

[2] E. Mostaani, N. D. Drummond, and V. I. Fal'ko. Quantum monte carlo calculation of the binding energy of bilayer graphene. *Phys. Rev. Lett.*, 115:115501, Sep 2015.

[3] Matthew Seymour and Nikolas Provatas. Structural phase field crystal approach for modeling graphene and other two-dimensional structures. *Phys. Rev. B*, 93(3), 2016.

[4] Songsong Zhou, Jian Han, Shuyang Dai, Jianwei Sun, and David J. Srolovitz. Van der waals bilayer energetics: Generalized stacking-fault energy of graphene, boron nitride, and graphene/boron nitride bilayers. *Phys. Rev. B*, 92(15), 2015.
